# Supplementary figures and images for: Dysfunction of Chloroplast Protease Activity Mitigates pgr5 Phenotype in the Green Algae Chlamydomonas reinhardtii
Source: Plants (Basel). 2024 Feb 23;13(5):606. doi: 10.3390/plants13050606 (PMC10935073; doi:10.3390/plants13050606)

# Figure S1

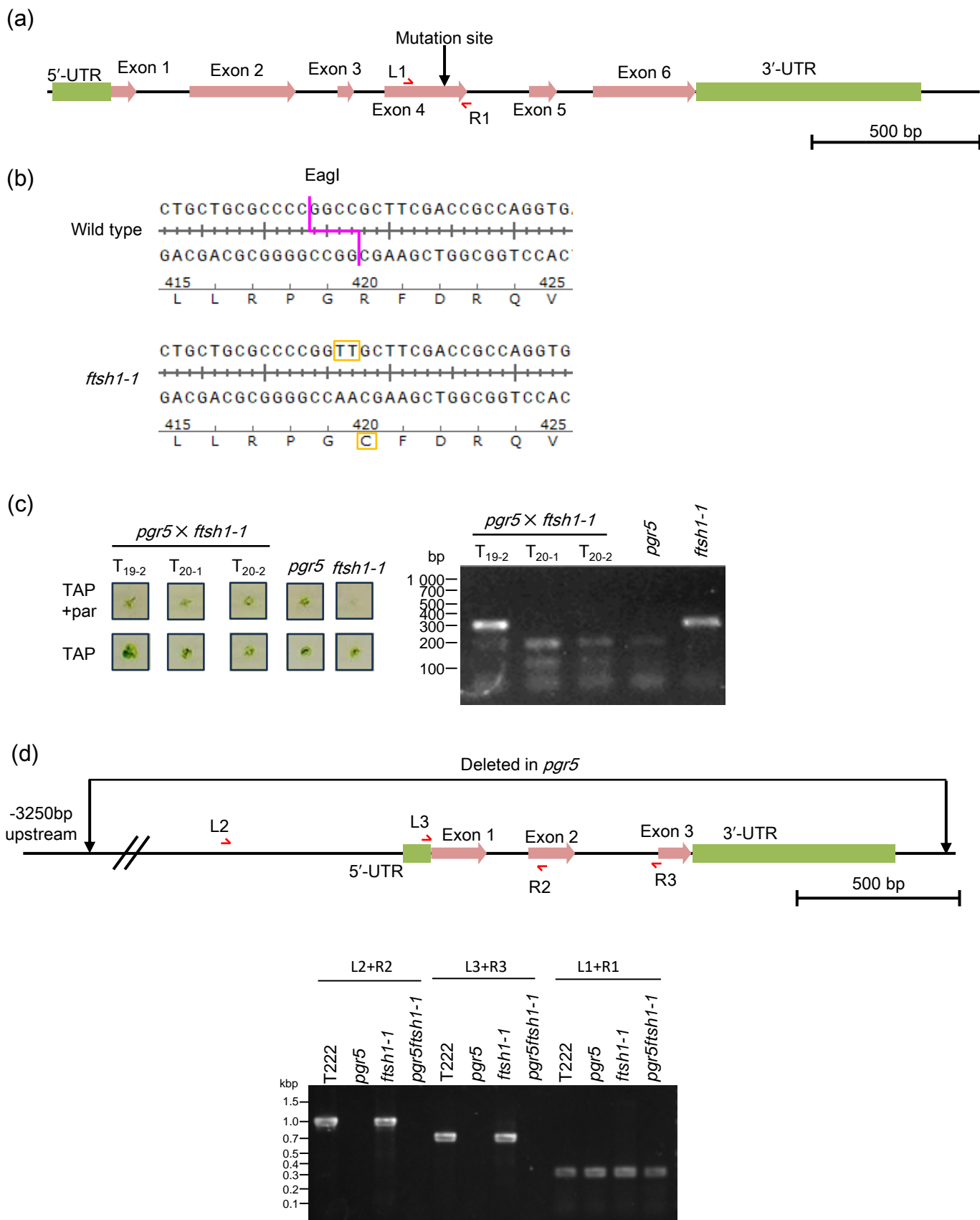

**Figure S2**

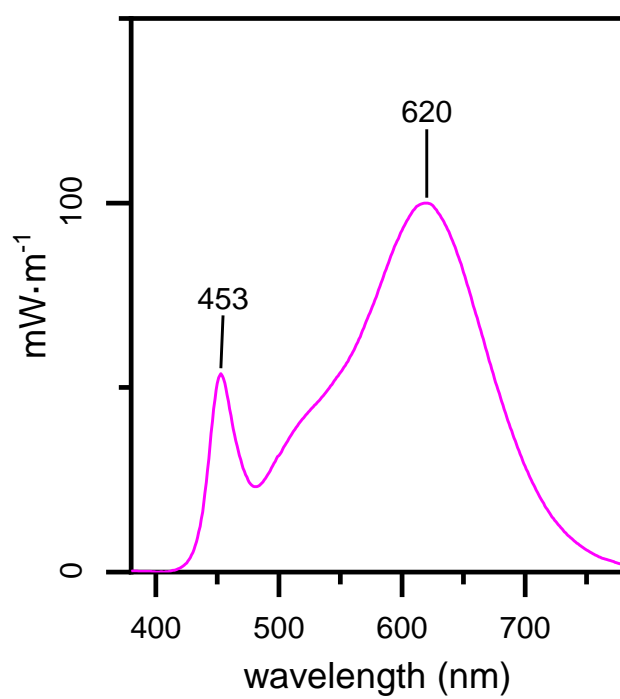

# Figure S3

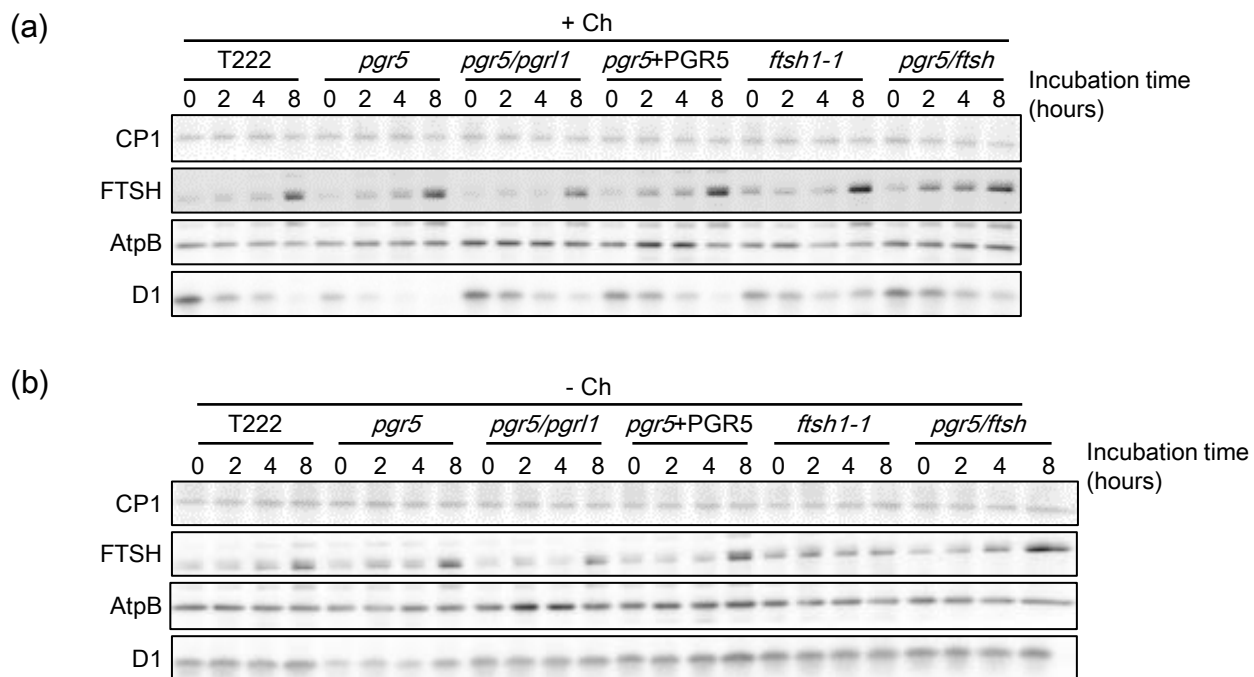

Supplement: Supplementary file 1 [file plants-13-00606-s001.zip › plants-2843372-supplementary.pdf]
